# Determinants of Newly-Diagnosed Raised Blood Pressure: The Malaysian Context

**DOI:** 10.21315/mjms2021.28.6.9

**Published:** 2021-12-22

**Authors:** Ooi Wei LIM, How Hui LIEW, Xin Ru ENG, Chin Khian YONG, Ling Hong LIM

**Affiliations:** 1 Malaysia Foundation Programme, Heriot-Watt University, Putrajaya, Malaysia; 2 Department of Mathematical and Actuarial Sciences, Lee Kong Chian Faculty of Engineering and Science, Universiti Tunku Abdul Rahman, Malaysia; 3 Department of Economics, School of Social Sciences, Heriot-Watt University, United Kingdom

**Keywords:** newly-diagnosed hypertension, sociodemographic determinants, behavioural determinants, dependency, raised blood pressure

## Abstract

**Background:**

Raised blood pressure, also known as hypertension (HPT), has been a distressing health concern among Malaysians. An upward trend is found on the prevalence of newly-diagnosed HPT, contributing to the high number of overall hypertensive patients in Malaysia. To understand the cause and reduce the economic burden caused by HPT, current research aims to examine the dependency among sociodemographic and behavioural determinants of newly-diagnosed HPT among Malaysians.

**Methods:**

The current study uses secondary data from the Fifth National Health and Morbidity Survey (NHMS V) 2015, a population based cross-sectional study. This study uses the Bayesian Network (BN) modelling to design and build a ‘causal’ model and identify potential determinants and their respective conditional probability on the prevalence of newly-diagnosed HPT among Malaysians.

**Results:**

This study shows that Malaysians with newly-diagnosed HPT are directly affected by the age and body mass index (BMI). Additionally, household income, sex, marital status, ethnicity, strata, education levels, occupation, fruit intake, vegetable intake, smoking status, physical activity and plain water intake indirectly affect the incidence of the newly-diagnosed HPT.

**Conclusion:**

These results may be helpful in implementing appropriate policies to prevent and monitor the increasing prevalence of newly-diagnosed HPT among adults in Malaysia.

## Introduction

Hypertension (HPT) is one of the non-communicable diseases (NCDs) that has prolonged conditions that does not result from an (acute) infectious process and hence is ‘not communicable’. HPT has been also recognised as a ‘silent killer’ because patients are unaware of any warning signs or symptoms (1). In addition, HPT is estimated to cause about 12.8% of the total annual death worldwide and is a major risk factor for coronary heart disease and stroke (2). Meanwhile, the direct cost to the Ministry of Health Malaysia for antihypertensive medication was known for RM570.3 million (USD141.33 million) in 2014 and RM608.8 million (USD150.87 million) in 2016 (3). Evidently, this economic burden for Malaysia will increase. If HPT is left untreated, it will lead to an increase in cardiovascular, cerebrovascular, and renal morbidity/mortality and overall mortality rate. Treating HPT for primary prevention is expected to have a higher impact than screening and treating HPT for secondary prevention (3). Besides, the prevalence of newly-diagnosed HPT will put an additional prevalence on the total number of hypertensive patients in Malaysia. Hence, investigating the behavioural and sociodemographic determinants among Malaysians is of the utmost importance to identify the potential predictors and assist in the early detection of newly-diagnosed HPT incidence in Malaysia.

The aim of this study is to identify the probabilistic relationship between the sociodemographic and behavioural determinants of newly-diagnosed HPT which will create insight for the government to strategise more effective intervention programmes in order to focus on dependency among the sociodemographic and behavioural determinants to monitor the newly-diagnosed HPT incidence among Malaysians. This study was carried out by employing the Bayesian Network (BN) model approach based on secondary data from the Fifth National Health and Morbidity Survey (NHMS V) 2015.

## Methods

### Data

Data used in this study are from the NHMS V 2015, which involved 19,936 respondents, age 18 years old and above, and among them 12,885 respondents who completed the survey in full reported that they were not told they had HPT by a doctor or assistant medical officer prior to the survey. The NHMS V 2015 is a population-based study that used two-stage stratified random sampling in which the primary stratum included the Federal Territories and states of Malaysia, while the second stratum consists of urban and rural strata. Those who stays in hotels, hostels, hospitals and so forth were excluded from the survey (4). Based on the NHMS V 2015 sampling frame provided by the Department of Statistics Malaysia, the geographical areas in Malaysia were divided into enumeration blocks (EBs). A total of 869 EBs were selected from the total EBs in Malaysia whereby 536 EBs were from urban areas and the remaining 333 EBs were from rural areas (4). Data were collected via two types of structured questionnaires — face-to-face interviews and self-administered — by the Institute for Public Health (IPH), Ministry of Health Malaysia.

### Variables

The targeted variables used in this study is newly-diagnosed HPT and the eligible sample cases ≥ 18 years old are 12,885 respondents. Newly-diagnosed HPT is defined as patients who are not known to have HPT and who have an average systolic blood pressure equal to or more than 140 mmHg and/or diastolic blood pressure greater than or equal to 90 mmHg (5). In accordance with the NHMS V 2015, participants’s blood pressure were taken by nurses. The validated and calibrated Omron Japan model HEM-907 was used for blood pressure assessment (5).

The behavioural determinants of this study include: body mass index (BMI) (underweight [BMI < 18.5 kg/m^2^]; normal [18.5 kg/m^2^ ≤ BMI < 25.0 kg/m^2^], overweight [BMI ≥ 25.0 kg/m^2^], obesity [BMI ≥ 30.0 kg/m^2^]) (6) and smoking status (current tobacco smoker, current non-smoker); physical activity (assessed by a short version of International Physical Activity Questionnaire; [active, inactive); fruit intake (adequate [≥ 2 units per day]; inadequate [≤ 2 units per day]); vegetable intake (adequate [≥ 3 units per day]; inadequate [≤ 3 units per day]) and plain water intake (adequate [≥ 6 glasses per day]; inadequate [≤ 6 glasses per day]) (7, 8).

The sociodemographic determinants included in the study are strata consisting of urban or rural state origin; sex (male or female); age group (years old) ([18–27], [28–37], [38–47], [48–57], [58–67], [68–77], [≥ 78]); marital status (never married, married, widow/widower/divorcee); ethnicity (Malay, Chinese, Indian, other Bumiputera, others); education level (no formal education, primary, secondary, tertiary, unclassified); occupation (government/semi government, private employee, self-employed, unpaid worker/homemaker, retiree); total household income and total monthly household expenditure.

### Statistical Model

The BNs are used to build models based on existing data and experts’ opinions which then predict the conditional probabilities of any possible contributing determinants in a study. A BN defines a joint probability distribution over a set of variables and the corresponding local univariate distribution. The directed acyclic graph associated with the BN determines whether each of them is marginal or conditional on other variables (9). The main role of the network structure is to express the conditional independence relationship between the variables in the model through graphical separation, thus specifying the factorisation of the global distribution (10):


(1) $$P(X)=\prod_{i=1}^{N}P(X_{i}|\pi_{X_{i}};\theta_{X_{i}})$$

where,

*X =* global probability distribution with parameters *θ*

*X_i_ =* random variable

*π_X_*_*_i_*_* =* parent of *X_i_*

Prior to the creat*io*n of BN model in this study, data cleaning occurred to remove any anomalies found so the dataset will be error-free and deliver accurate and complete information. A clean dataset will boost the decision-making process and enable the algorithm to provide a better prediction (11).

Designing a BN model manually requires a significant amount of effort (12). Therefore, hill-climbing (HC) algorithm has been used in this study to simplify the modelling process. HC algorithm is a type of score-based algorithms that explores the dataset and creates an empty directed acyclic graph (DAG) with no arcs, and, it adds, deletes or reverses one arc at a time and chooses the adjustment that increases the network score until the score can no longer be improved (9). Random restarts were enabled in the HC algorithm to avoid local optima. Additionally, the algorithm was optimised by using score caching, score decomposability and score equivalence to reduce the number of duplicated tests (13).

Network score is a type of goodness-of-fit statistics that measures how well the DAG mirrors the dependence structure of the data (9). Bayesian Information Criterion (BIC) is the default network scoring criterion in the HC algorithm, *hc* function included in the bnlearn package. The BIC statistic is defined as:


(2) BIC=ln (N)*k-2*LL(2)

where *In*( ) is the natural logarithm, *LL* is the log-likelihood of the model, *N* is the sample size of dataset and *k* is the number of parameters in the model (14). Network score evaluation and model adjustment will be carried out until the score can no longer be improved. The statistical analyses have been conducted using bnlearn (R package).

**Figure 1:**
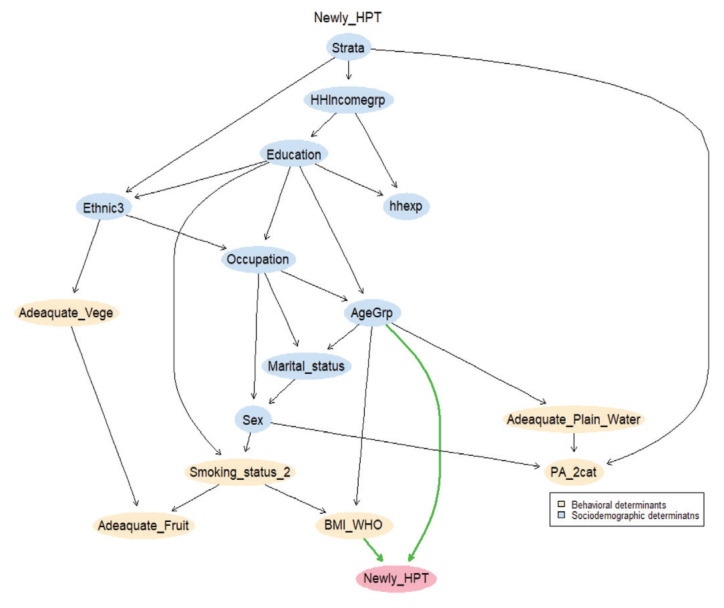
Conditional probabilities of newly-diagnosed HPT

In this study, BN has been employed to estimate the conditional probability of newly-diagnosed HPT because it can better reveal the relationship between the determinants and newly-diagnosed HPT. The structure of BN is represented by DAG, in which the nodes represents determinants and the edges express the dependencies between the determinants. Therefore, the parameter indicates the relationship between a node and its parent nodes on the DAG. Parameter learning has been used to calculate the conditional probability distribution of each node based on secondary data and given structure. HC has been recognised as one of structural algorithms to be employed to build the BN model by using bnlearn (R package). The probabilistic statistical measures showed both lower Akaike Information Criteria (AIC) values and also BIC values, indicating a better fit model (AIC= −317676.9, BIC= −320167.8) as shown in Figure 1 (15, 16).

## Results

### Descriptive Statistics Among Newly-Diagnosed Hypertension Respondents

A total of 12,885 adults were included in this study but the known hypertensive respondents were not considered. The characteristics of newly-diagnosed HPT respondents are shown in Table 1. Of the total respondents, 23.81% were newly diagnosed with HPT. The percentage of newly-diagnosed HPT incidence was higher among males (12.32%) than females (11.49%). The 48–57-year-old age group exhibited the highest incidence of newly-diagnosed HPT (6.87%), followed by the 38–47-year-old age group (5.79%), then the 58–67-year-old age group (4.13%), 28–37-year-old age group (3.97%), 18–27-year-old age group (1.83%), 68–77-year-old age group (1.07%), and finally 78-year-old and above age group (0.16%), respectively. The majority of the newly-diagnosed HPT incidence was found among respondents with secondary education (11.27%), inadequate fruit intake (21.44%), inadequate vegetable intake (21.20%), adequate plain water intake (18.19%) and a household income between RM1,000–RM1,999 (4.98%). Next, married respondents (19.00%), private employees (7.10%), overweight respondents (9.15%), physically active respondents (17.90%), current non-smokers (18.40%) and urban dwellers (12.79%) exhibited high percentages of newly-diagnosed HPT. By ethnicity, the highest incidence of newly-diagnosed HPT was found among Malays (15.94%), followed by Chinese (3.10%), others Bumiputera (1.92%), others (1.47%), and Indian (1.38%), respectively.

### Inference of Bayesian Network for Interaction Between Body Mass Index and Age Group on Newly-Diagnosed Hypertension

In this learned network (Figure 1), the behavioural determinant, BMI, and the sociodemographic determinant, age group, were parents of newly-diagnosed HPT, which influences the newly-diagnosed HPT incidence among Malaysians.

Based on the BN model, the network allows us to identify the ‘parent’ determinants, BMI and age group, that directly influenced the conditional probability of developing newly-diagnosed HPT when under the stated condition both by the model. The conditional probability table (CPT) (Table 2) indicated that the interaction between age and BMI is positive; the incidence of HPT increases with BMI and ages, showing that the obese respondents have the highest conditional probability of newly-diagnosed HPT when they are 78 years old and above (50%). Hence, Table 2 shows that the conditional probabilities of getting newly-diagnosed HPT increase as the BMI increases at any age level, and the conditional probabilities of getting newly-diagnosed HPT are lower when a person is younger, and are higher as a person ages. Similarly, the conditional probabilities of getting newly-diagnosed HPT increase as a person gets older at any BMI level, and they are lower when a person’s BMI is lower and are higher when a person’s BMI is high or obese. As a result, this study revealed that the conditional probability of newly-diagnosed HPT rises as BMI and age group increases.

### Inference of Bayesian Network of Behavioural Determinants on Newly-Diagnosed Hypertension

However, the conditional probability of newly-diagnosed HPT distributions in the model were dependent on its structure as well as on the data. Based on Figure 1, BMI is directly leads to the occurrence of newly-diagnosed HPT as per discussed above.

The existence of other determinants are also considered in the occurrence of newly-diagnosed HPT among Malaysians. The BN allows researchers to elicit the structure of the determinants and identify ‘parent’ variables that directly influenced other variables. Smoking status is indirectly link to the occurrence of newly-diagnosed HPT as shown in Figure 1. Table 3 shows the conditional probability of developing newly-diagnosed HPT was 20.9% among current non-smoker respondents, compared to 19.0% among current tobacco smokers. In addition, respondents with adequate plain water intake showed higher conditional probability of newly-diagnosed HPT incidence (20.57%) compared to inadequate plain water intake (19.94%). Physically inactive respondents demonstrate higher occurrence of newly-diagnosed HPT incidence (20.44%) compared to physically active respondents (20.42%). This study shows a higher conditional probability of newly-diagnosed HPT incidence among respondents with adequate fruit intake (20.57%) compared to inadequate fruit intake (20.40%). Finally, the CPT demonstrates higher conditional probability of newly-diagnosed HPT incidence among respondents with adequate vegetable intake (20.51%) compared to inadequate vegetable intake (20.40%).

### Inference of Bayesian Network of Sociodemographic Determinants on Newly-Diagnosed Hypertension

According to Figure 1, age has been observed to be the ‘parent’ node and directly link to occurrences of newly-diagnosed HPT. Among the age groups, the highest conditional probability of newly-diagnosed HPT was 30.56% for those above 78 years old, 29.91% for those between 58 years old and 67 years old, 28.22% for those between 48 years old and 57 years old, 26.98% for those between 68 years old and 77 years old, 22.31% for those between 38 years old and 47 years old, 14.57% for those between 28 years old and 37 years old, 8.84% for those between ages 18 years old and 27 years old, respectively, as shown in Table 2.

Female respondents have a higher occurrence of newly-diagnosed HPT incidence (21.28%) than the male respondents (19.58%). This result is differs from the percentage obtained in the descriptive statistics; this reversed result is because of other determinants in the BN. Because the conditional probability of newly-diagnosed HPT was calculated based on the relationships between the determinants and newly-diagnosed HPT. This indicated that the BN model would be able to predict the interrelationships among the sociodemographic and behavioural determinants which would lead to the occurrence of newly-diagnosed HPT incidence. Widow/widower/divorcees (25.64%) were observed to have a higher occurrence of newly-diagnosed HPT incidence compared to married (22.02%) and never married respondents (12.17%). Among the retirees, the conditional probability of newly-diagnosed HPT incidence was the highest (28.52%), followed by government/semi-government workers (19.58%), unpaid workers/homemakers (23.15%), self-employed (21.86%) and private employees (17.28%). Other Bumiputera (21.14%) reflects the highest conditional probability of newly-diagnosed HPT incidence among the ethnics, followed by Chinese (20.52%), Malays (20.37%), others (20.35%) and Indian (20.01%). The conditional probability of newly-diagnosed HPT incidence was highest among respondents with primary education (24.33%), followed by those with no formal education (23.64%), secondary education (19.81%), tertiary education (17.59%) and unclassified education (16.53%). The condition probability of newly-diagnosed HPT incidence is also indirectly affected by household income among the respondents. Lower household income earners reflected higher condition probability of newly-diagnosed HPT incidence. Households with incomes below RM1,000 showed 21.88%, followed by households with incomes between RM1,000 and RM1,999 (21.25%), households with incomes between RM2,000 and RM2,999 (20.74%), households with incomes between RM3,000 and RM3,999 (20.48%), households with incomes between RM4,000 and RM4,999 (20.07%), households with incomes between RM5,000 and RM5,999 (19.80%), households with incomes between RM6,000 and RM6,999 (19.54%), households with incomes between RM7,000 and RM7,999 (19.50%), households with incomes between RM8,000 and RM8,999 (19.38%), households with incomes between RM9,000 and RM9,999 (18.90%) and finally households with income RM10,000 and above (18.72%). Lastly, rural dwellers exhibited a higher condition probability of newly-diagnosed HPT incidence (20.82%) compared to urban dwellers (20.18%).

## Discussion

Based on the results from the inference of the selected BN model (Figure 1) on the determinants leading to the occurrence of newly-diagnosed HPT incidence, when meeting the age group and BMI condition, the results show an increased predictive risk of newly-diagnosed HPT incidence when both BMI and age increased among the respondents in Malaysia. Therefore, these findings imply that increased weight leads to increased newly-diagnosed HPT (17, 18). Moreover, as people get older, their arteries may become less elastic, which may contribute to the development of newly-diagnosed HPT (19). However, BMI is modifiable and people can act to reduce the incidence of newly-diagnosed HPT.

### Behavioural Determinants on Newly-Diagnosed Hypertension

Based on the structure and data of the BN model, the conditional probability of developing newly-diagnosed HPT incidence revealed the highest occurrence of newly-diagnosed HPT (30.56%). However, it was lower than the conditional probability of newly-diagnosed HPT (50.0%) when the network was given both condition which includes BMI and age group concurrently. These results indicate it is essential for policy makers to identify intervention targets by looking into the maintenance of normal body weight among Malaysians, especially the middle aged and elderly, because excess weight gain tends to contribute to the development of insulin resistance and increased blood pressure (20).

Figure 1 shows that the BN model provides indirect pathways of influence on the conditional probability of newly-diagnosed HPT incidence because it can identify which behavioural determinants are directly and indirectly linked to the occurrence of newly-diagnosed HPT, which could suggest potential intervention targets to control and prevent the occurrence of newly-diagnosed HPT. Figure 1 also shows that smoking status correlates with BMI. Table 3 shows the probability of developing newly-diagnosed HPT incidence was higher among current non-smoker respondents.

Table 3 shows that the conditional probability of developing newly-diagnosed HPT incidence was 20.9% among current non-smoker respondents compared to 19.0% among current tobacco smokers. This unexpected results may be due to some ex-smokers who are included in the current non-smokers database. This also can be supported by a previous study which indicated that ex-smokers had significantly higher odds of known HPT and reported that regular and long cigarette smoking was associated with HPT (21–23).

Next, the results among the respondents with adequate plain water intake showed higher conditional probability of newly-diagnosed HPT incidence (20.57%). This is supported by a previous study which indicated there was no statistically significant differences in the prevalence of HPT between people drinking more or less than the daily recommended amount of water (24). There was a higher occurrence of newly-diagnosed HPT incidence (20.45%) among physically inactive respondents, which is consistent with a previous research that revealed that physical inactivity was significantly associated with increased odds of newly-diagnosed HPT among urban Chinese adults (25). Next, respondents with adequate fruit intake (20.57%) demonstrate higher conditional probability of newly-diagnosed HPT than those with an inadequate fruit intake (20.40%) which may due to sampling error and the effect may not be significant. It may be concluded that fruit intake may not affect newly-diagnosed HPT.

Additionally, this study demonstrates a slightly higher conditional probability of newly-diagnosed HPT incidence among respondents with adequate vegetable intake (20.51%) compared to inadequate vegetable intake (20.40%). However, the difference is not large enough to conclude that the adequate vegetables intake will affect the incidence of newly-diagnosed HPT. Hence, this study also reflects certain eating habits, for instance vegetarians may be not less prone to getting newly diagnosed HPT.

As a result, BN provides insights that is related to which behavioural determinants are directly or indirectly predicting the newly-diagnosed HPT incidence among Malaysians.

### Sociodemographic Determinants on Newly-Diagnosed Hypertension

The conditional probability of developing newly-diagnosed HPT is found higher among female respondents, which supports a previous study which stated that females have a 39.3% prevalence which is slightly higher than the males 36.7% incidence of newly-diagnosed HPT in males (17). Additionally, widow/widower/divorcees are observed to have a higher occurrence of newly-diagnosed HPT incidence (25.64%) and this outcome is consistent with a previous research which reported that married individuals have potentially greater financial resources available for health care and for promoting a healthier lifestyle (27). The retirees have reflected the highest conditional probability of newly-diagnosed HPT incidence (28.52%), and this finding supports a previous research outcome which stated that employment status was not a significant risk factor for HPT (28) because the BN model elicits occupation as one of the indirect determinants leading to the occurrence of newly-diagnosed HPT among Malaysians as shown in Figure 1.

Among the ethnicity groups, other Bumiputera (21.35%) has the highest conditional probability of newly-diagnosed HPT incidence which matches a previous study that found other Bumiputera were 1.55 times more likely to have HPT when compared to Malays (29). This study reveals that the conditional probability of newly-diagnosed HPT is higher among respondents with primary education (24.33%) and it is indirectly linked to the occurrence of newly-diagnosed HPT incidence, which is consistent with previous research found that those with a lower educational status or those who are illiterate were observed to have a 34.9% prevalence in newly-diagnosed HPT (30).

This study demonstrates that lower household income earners have a higher condition probability on newly-diagnosed HPT incidence, which supports previous research which stated that respondents with higher income had lower odds (odds ratio = 0.71; 95% confidence interval = 0.56, 0.91) of being newly-diagnosed HPT (25). It is suggested that higher income group respondents would have better access to medical facilities; for example, health screening to monitor blood pressure, which would reduce the conditional probability of newly-diagnosed HPT among adults in Malaysia.

Finally, rural dwellers exhibited higher conditional probability of newly-diagnosed HPT incidence compared to urban dwellers which supports a previous researcher who stated that the newly-diagnosed HPT rate was significantly higher in rural areas than in urban areas (31). The probable reason for this result is that people who stay in urban areas have better access to healthcare and are more likely to be treated (32).

## Conclusion

In this study, BN was employed to study the incidence of newly-diagnosed HPT with relevant sociodemographic and behavioural determinants and to explore the relationship between the sociodemographic and behavioural determinants to predict the incidence of newly-diagnosed HPT among Malaysians. Different combinations of the sociodemographic and behavioural determinants can significantly increase the probability of newly-diagnosed HPT incidence. Identifying the relationship between the sociodemographic and behavioural determinants on newly-diagnosed HPT will create insight in order for the government to develop more effective intervention programmes in order to focus on dependency among the sociodemographic and behavioural determinants to monitor the newly-diagnosed HPT incidence among Malaysians.

## Figures and Tables

**Table 1:** Descriptive statistics among newly-diagnosed HPT respondents

Variables	Levels	No HPT frequency (%)	Newly-diagnosed HPT frequency (%)
Gender	Male	4,975 (38.61)	1,588 (12.32)
	Female	4,841 (37.57)	1,481 (11.49)
Age group (years old)	0 = 18–27	2,399 (18.62)	236 (1.83)
	1 = 28–37	2,861 (22.20)	511 (3.97)
	2 = 38–47	2,216 (17.20)	746 (5.79)
	3 = 48–57	1,533 (11.90)	885 (6.87)
	4 = 58–67	625 (4.85)	532 (4.13)
	5 = 68–77	164 (1.27)	138 (1.07)
	6 = 78 and above	18 (0.14)	21 (0.16)
Body mass index (BMI)	Underweight	676 (5.25)	44 (0.34)
	Normal	4,790 (37.18)	900 (6.98)
	Overweight	2,902 (22.52)	1,179 (9.15)
	Obese	1,448 (11.24)	946 (7.34)
Educational level	Unclassified	113 (0.88)	25 (0.19)
	No formal education	347 (2.69)	162 (1.26)
	Primary education	1,705 (13.23)	885 (6.87)
	Secondary education	4,965 (38.53)	1,452 (11.27)
	Tertiary education	2,686 (20.85)	545 (4.23)
Ethnicity	Malay	5,949 (46.17)	2,054 (15.94)
	Chinese	1,526 (11.84)	400 (3.10)
	Indian	699 (5.42)	178 (1.38)
	Others	800 (6.21)	189 (1.47)
	Others Bumiputera	842 (6.53)	248 (1.92)
Household income	Less than RM1,000	940 (7.30)	433 (3.36)
	RM1,000–RM1,999	1,627 (12.63)	642 (4.98)
	RM2,000–RM2,999	1,716 (13.32)	597 (4.63)
	RM3,000–RM3,999	1,394 (10.82)	380 (2.95)
	RM4,000–RM4,999	998 (7.75)	278 (2.16)
	RM10,000 and above	797 (6.19)	203 (1.58)
	Other	2,344 (18.19)	536 (4.16)
Marital status	Married	6,997 (54.30)	2,448 (19.00)
	Never married	2,339 (18.15)	351 (2.72)
	Widow/Widower/Divorce	480 (3.73)	270 (2.10)
Occupation	Government/Semi-government	1,379 (10.70)	402 (3.12)
	Private employee	4,242 (32.92)	915 (7.10)
	Retiree	261 (2.03)	185 (1.44)
	Self-employed	2,262 (17.56)	884 (6.86)
	Unpaid worker/homemaker	1,672 (12.98)	683 (5.30)
Physical activity	Active	7,213 (55.98)	2,307 (17.90)
	Inactive	2,603 (20.20)	762 (5.91)
Smoking status	Current tobacco smoker	2,613 (20.28)	698 (5.42)
	Current non-smoker	7,203 (55.90)	2,371 (18.40)
Strata	Urban	5,999 (46.56)	1,648 (12.79)
	Rural	3,817 (29.62)	1,421 (11.03)
Fruit intake	Adequate	1,010 (7.84)	307 (2.38)
	Not adequate	8,806 (68.34)	2,762 (21.44)
Vegetable intake	Adequate	1,107 (8.59)	338 (2.62)
	Not adequate	8,709 (67.59)	2,731 (21.20)
Plain water intake	Adequate	7,171 (55.65)	2,344 (18.19)
	Not adequate	2,645 (20.53)	725 (5.63)

**Table 2:** CPT of newly-diagnosed HPT on BMI and age group

Newly-diagnosed HPT	BMI WHO	Age group						

		0	1	2	3	4	5	6
Yes	Underweight	0.0181	0.0303	0.0804	0.1613	0.1429	0.2143	0.4000
Yes	Normal	0.0492	0.0845	0.1451	0.2394	0.2741	0.2780	0.2927
Yes	Overweight	0.1236	0.1500	0.2419	0.2989	0.3262	0.2599	0.3529
Yes	Obese	0.2344	0.2836	0.3403	0.3302	0.3075	0.3016	0.5000

Notes: Age group indicator: 0 = 18–27 years old; 1 = 28–37 years old; 2 = 38–47 years old; 3 = 48–57 years old; 4 = 58–67 years old; 5 = 68–77 years old; 6 = 78 years old and above

**Table 3:** CPT of newly-diagnosed HPT on respective determinants

Variable	Levels	Conditional probability
Plain water intake	Adequate	0.19944
	Inadequate	0.20571
Vegetable intake	Adequate	0.20509
	Inadequate	0.20404
Physical activity	Active	0.20416
	Inactive	0.20447
Education	No formal education	0.23638
	Primary	0.24334
	Secondary	0.19808
	Tertiary	0.17594
	Unclassified	0.16532
Ethnicity	Chinese	0.20516
	Indian	0.20011
	Malay	0.20373
	Others	0.20352
	Others Bumiputera	0.21135
Household income	Less than RM1,000	0.21879
	RM1,000–RM1,999	0.21252
	RM2,000–RM2,999	0.20738
	RM3,000–RM3,999	0.20481
	RM4,000–RM4,999	0.20075
	RM5,000–RM5,999	0.19799
	RM6,000–RM6,999	0.19540
	RM7,000–RM7,999	0.19506
	RM8,000–RM8,999	0.19384
	RM9,000–RM9,999	0.18899
	RM10,000 and above	0.18721
Fruit intake	Adequate	0.20574
	Inadequate	0.20401
Marital status	Married	0.22023
	Never married	0.12172
	Widow/Widower/Divorcee	0.25638
Occupation	Government/Semi-government	0.19576
	Private employee	0.17279
	Retiree	0.28519
	Self-employed	0.21858
	Unpaid worker/Homemaker	0.23149
Sex	Female	0.21276
	Male	0.19579
Smoking status	Current non-smoker	0.20897
	Current tobacco smoker	0.19029
Strata	Rural	0.20822
	Urban	0.20179
BMI	Underweight	0.05953
	Normal	0.14607
	Overweight	0.23602
	Obese	0.30561

Notes: By using this model, the network relationship showed the related parameters of the related behavioural and sociodemographic determinants on the occurrence of newly-diagnosed HPT incidence. The higher conditional probability will indicate higher occurrence of the newly-diagnosed HPT
